# UK clinical practice guidelines for the management of gastrointestinal stromal tumours (GIST)

**DOI:** 10.1186/s13569-017-0072-8

**Published:** 2017-04-21

**Authors:** Ian Judson, Ramesh Bulusu, Beatrice Seddon, Adam Dangoor, Newton Wong, Satvinder Mudan

**Affiliations:** 1 The Institute of Cancer Research, Royal Marsden NHS Foundation Trust, Fulham Road, London, SW3 6JJ UK; 2 Addenbrooke’s Hospital, Cambridge University Hospitals, Cambridge, UK; 3 University College Hospital, University College London Hospitals NHS Foundation Trust, London, UK; 4 Bristol Cancer Institute, University Hospitals, Bristol NHS Trust, Bristol, UK; 5 Southmead Hospital, North Bristol NHS Trust, Bristol, UK; 6 Royal Marsden NHS Foundation Trust London, London, UK

## Abstract

**Background:**

Soft tissue sarcomas (STS) are rare tumours arising in mesenchymal tissues. Gastrointestinal stromal tumour (GIST) is the commonest STS and arises within the wall of the gastrointestinal (GI) tract. While most GISTs occur in the stomach they do occur in all parts of the GI tract. As with other STS, it is important that GISTs are managed by expert teams, to ensure consistent and optimal treatment, as well as recruitment to clinical trials, and the ongoing accumulation of further knowledge of the disease. The development of appropriate guidance, by an experienced panel referring to the evidence available, is therefore a useful foundation on which to build progress in the field.

**Methodology:**

British Sarcoma Group guidelines for the management of GIST were initially developed by a panel of physicians experienced in the management of GIST. This current version has been updated and amended with reference to other European and US guidance. We have received input from representatives of all diagnostic and treatment disciplines as well as patient representatives. Levels of evidence and strength of recommendation gradings are those used by ESMO adapted from those published by the Infectious Disease Society of America.

**Conclusions:**

The guidelines cover aetiology, genetics and underlying molecular mechanisms, diagnosis and initial investigations, staging and risk stratification, surgery, neoadjuvant and adjuvant therapy, the management of advanced disease and follow-up. The importance of mutational analysis in guiding treatment is highlighted, since this can indicate the most effective treatment and avoid administration of ineffective drugs, emphasising the need for management in specialist centres.

## Background

British Sarcoma Group guidelines for the management of GIST were initially developed in 2004 by a panel of physicians experienced in the management of GIST representing all diagnostic and treatment disciplines. These Clinical Practice Guidelines update reflect the improvements in our knowledge of the disease and developments that have taken place since then [1]. Levels of evidence and strength of recommendation gradings are those used by ESMO adapted from those published by the Infectious Disease Society of America (Table 1) [2].

**Table 1:** Levels of evidence and Grades of recommendation

*Levels of evidence*
I Evidence from at least one large randomised, controlled trial of good methodological quality (low potential for a bias) or meta-analyses of well-conducted randomised trials without heterogeneity
II Small randomised trials or large randomised trials with a suspicion of bias (lower methodological quality) or meta-analyses of such trials or of trials with demonstrated heterogeneity
III Prospective cohort studies
IV Retrospective cohort studies or case–control studies
V Studies without control group, case reports, and experts’ opinions
*Grades of recommendation*
A Strong evidence for efficacy with a substantial clinical benefit, strongly recommended
B Strong or moderate evidence for efficacy but with a limited clinical benefit, generally recommended
C Insufficient evidence for efficacy or benefit does not outweigh the risk or the disadvantages (adverse events, costs,…), optional
D Moderate evidence against efficacy or for adverse outcome, generally not recommended
E Strong evidence against efficacy or for adverse outcome, never recommended

## Incidence

Gastrointestinal stromal tumours (GISTs) are rare cancers, with an estimated unadjusted incidence of 1.5/100,000/year [3]. Data from the Rhȏne-Alpes region of France [4] and “NHS England Cancer Registry” (personal communication) suggest an incidence of just under 11 per million per annum, equating to 650 clinically meaningful new cases a year in the UK, approximately 900 in total. Accurate data on prevalence in the UK are not yet available.

The median age at diagnosis is around 60–65 years, with a wide range. Occurrence in children, adolescents and younger patients is very rare, although paediatric GISTs represent a distinct subset, marked by female predominance, absence of *KIT*/platelet-derived growth factor alpha (*PDGFRA*) mutations, usually gastric origin, often multifocal, and with possible lymph node metastases [5].

## Aetiology

In most cases the aetiology is unknown, although it is reported that patients with GIST are more likely to be diagnosed with another cancer than the general population [6, 7], suggesting a likely link with inherited increased susceptibility to cancer in some patients. In the majority of cases GIST is associated with an activating mutation in either the *KIT* or *PDGFRA* (platelet derived growth factor receptor alpha) gene. However, other rare drivers may include mutations in *NF1 (*neurofibromatosis type 1, loss of function) or *BRAF* (gain of function). Tumours lacking mutations in *KIT* or *PDGFRA* are often called “wild-type”, and those lacking mutations in not only these genes but also *BRAF* and *NF1* have been dubbed “quadruple wild-type” [8]. Specific advice concerning the management of patients with paediatric, “wild-type” and syndromic GIST can be obtained from a UK based alliance of medical specialists via the web site http://www.pawsgistclinic.org.uk.

A number of genetic syndromes are linked to GIST:

- The Carney triad syndrome, comprising gastric GIST, paraganglioma and pulmonary chondroma (these may occur at different ages) [9].
- Carney–Stratakis syndrome, marked by germ-line mutations of one of the SDH subunits A, B, C or D, leading to a dyad of GIST and paraganglioma [10, 11].
- Type-1 neurofibromatosis, i.e. associated with loss of function of *NF1,* whether sporadic or inherited, and absence of mutations in *KIT or PDGFRA*, the GISTs are often multifocal, predominantly located in the small bowel [12].
- Familial GIST, i.e. families with a germ-line autosomal dominant mutation of *KIT,* are extremely rare, presenting with multiple GISTs at an early age.

## Diagnosis

### Clinical presentation and investigation

The most common symptoms of GIST include upper gastrointestinal bleeding and anaemia, whilst larger tumours may present with abdominal pain/discomfort and a palpable mass. Small bowel GISTs may remain silent for a long period before presenting with an acute event such as haemorrhage or rupture. Symptomatic colorectal GISTs may present with abdominal pain, obstruction and lower gastrointestinal bleeding; oesophageal and gastro-oesophageal junction GISTs with dysphagia. Some patients may have non-specific systemic symptoms such as weight loss, night sweats and fever. Lack of awareness of the presenting features may lead to delayed diagnosis of GIST in some patients. Small GISTs may be asymptomatic and are often diagnosed incidentally during investigation for other conditions.

Small lesions below 2 cm in diameter may safely be followed by endoscopic ultrasound on an annual basis and biopsied or excised if they continue to grow. For larger lesions it is necessary to make a histological diagnosis. The standard approach for small nodules of 2 cm or above is excisional biopsy, as GISTs of this size carry a higher risk. For gastric lesions it is preferable to perform a fine needle aspirate or core needle biopsy under endoscopic ultrasound (EUS) guidance. If this is not feasible, and limited surgery is possible, primary resection may be appropriate. However, the differential diagnosis of intra-abdominal tumours may include leiomyosarcoma, germ cell tumour, lymphoma, benign and malignant neurogenic tumours, and fibromatosis. Given that the management of these conditions differs substantially, and primary excision is not always appropriate, it is sometimes necessary to do a percutaneous core needle biopsy to confirm the diagnosis. This however carries a very small risk of contaminating the peritoneal cavity, especially if bleeding were to occur. If surgery would require multi visceral resection, or is likely to be morbid, e.g. total gastrectomy, then multiple core needle biopsies are definitely required, again ideally under EUS guidance, or alternatively using an ultrasound/computed tomography (CT)-guided percutaneous approach. Depending on the histological diagnosis initial treatment may be with systemic therapy, e.g., lymphomas, mesenteric fibromatosis, germ cell tumours and GIST, or alternatively surveillance for benign entities such as non-malignant neurogenic tumours. Lesions at risk of rupture, such as cystic masses, should only be biopsied in specialised centres. If a patient presents with obvious metastatic disease, then a biopsy of an easily accessible metastatic focus should be performed and a laparotomy for diagnostic purposes is usually unnecessary.

Pathologically, the diagnosis of GIST relies on morphological assessment and immunohistochemistry, the diagnosis being supported by CD117 immunopositivity [13, 14]. More recently DOG1 has been added to the diagnostic armamentarium [15, 16]. About 5% of GISTs are CD117 immunonegative, about 5% of GISTs are DOG1 immunonegative and about 1% of GISTs are immunonegative for both. The mitotic count has prognostic value and although several risk assessment tools use an index of mitoses per 50 high-power fields, it would be more accurate and reproducible to express this as the number of mitoses in a total area of 5 mm^2^, which is therefore recommended. If there is some diagnostic doubt, particularly in CD117 and/or DOG1 immunonegative suspected GIST, molecular analysis for activating mutations in *KIT* or *PDGFRA* may help confirm the diagnosis. Mutational analysis has predictive value for sensitivity to molecular-targeted therapy, and also prognostic value. If initial treatment is with imatinib, mutational analysis is particularly critical, since some GISTs are insensitive to the drug (e.g. *PDGFRA* exon 18 mutation D842V). The inclusion of mutational analysis in the diagnostic work-up of all GISTs should be considered standard practice, with the possible exclusion of sub 2 cm non-rectal GISTs, which are very unlikely ever to need medical treatment. It is strongly recommended that mutational analysis is performed in centralised laboratories which are enrolled in an external quality assurance program, and which have expertise in GIST analysis. In *KIT/PDGFRA* mutation negative, or “Wild-Type” GIST, immunohistochemistry for succinate dehydrogenase B (SDHB), and, if negative, SDHA, should be performed if available, since loss of expression may assist the diagnosis and may help to guide therapy. In the absence of mutations in *KIT* or *PDGFRA* it is also important to look for mutations in *BRAF,* a rare, but important finding from a treatment perspective, since BRAF inhibitors are available, and also in *NF1.* Patients with neurofibromatosis, who have a germline mutation in *NF1,* are at increased risk of GIST and this finding may indicate occult neurofibromatosis. The optimal treatment for advanced GIST with *NF1* mutation has yet to be determined. Collection of fresh/frozen tissue is encouraged, because new molecular pathology assessments can then be made at a later stage in the patient’s interest. Informed consent for tumour banking should be sought, so that the tissue is available for later analyses and research, provided that local ethical approval is in place. Consent forms for the National GIST Tissue Bank based at the Royal Marsden Hospital can be found at http://www.givemysample.org/gist.

#### Key recommendations:

1. Lesions larger than 2 cm in diameter need to be investigated and a diagnosis made. Often this can be done by endoscopic ultrasound and needle biopsy, particularly if the lesion is in the stomach.
2. The diagnosis should be made by a pathologist experienced in the disease and include the use of immunohistochemistry and mutational analysis, which should be performed by an accredited laboratory.
3. If neoadjuvant treatment with imatinib is planned, it is vital to confirm the diagnosis, since there is a wide differential. It may be necessary to perform a percutaneous core needle biopsy if the tumour is inaccessible to endoscopic ultrasound-guided biopsy. Mutational analysis is obligatory, since some GISTs are insensitive to imatinib (e.g. those with D842V mutation in exon 18 of *PDGFRA*).

## Risk assessment for primary tumours with no evidence of metastatic disease

The TNM classification for staging has several limitations and its use is not recommended in this disease. Prognostic factors of proven value are the mitotic rate, tumour size, tumour site, and presence or absence of tumour rupture. Gastric GISTs have a better prognosis than small bowel or rectal GISTs. Tumour rupture through a serosal surface is an adverse prognostic factor and should be recorded, whether it took place before or during surgery. Mutational status has not been incorporated in any risk classification so far, although some genotypes have a distinct natural history, for example *KIT/PDGFRA* wild type GISTs tend to exhibit more indolent behaviour than *KIT* exon 11 mutant disease.

It is possible to assess risk of recurrence after resection of a localised GIST. This can be especially useful in deciding the role of adjuvant therapy in individual patients. Several risk classifications have been proposed. An initial risk classification developed at a consensus meeting convened by the National Institute of Health (NIH) [13] was useful but only considered size and mitotic index. Although the ‘high-risk’ category based on the NIH criteria has a much worse prognosis than the others, with both ‘very low-risk’ and ‘low-risk’ categories having a very favourable prognosis, the ‘intermediate-risk’ category did not reliably identify patients with an unfavourable prognosis. A risk classification was proposed by the Armed Forces Institute of Pathology, which incorporates the primary tumour site, in addition to mitotic count and tumour size, i.e. three of the main prognostic factors in localized GISTs [17]. There are also problems with the application of this risk classification tool since mitotic index and tumour size are non-linear continuous variables, so that the risk thresholds, especially based on number of mitoses, need to be interpreted with care. A modification to the NIH criteria has been proposed that incorporates tumour site and additionally tumour rupture, an important risk factor, [18] see Table 2. Prognostic contour maps have been generated through a number of series of GIST patients not treated with adjuvant therapy. These maps incorporate the mitotic index and tumour size as continuous non-linear variables, while tumour rupture is considered in addition to tumour site. They have been validated against pooled data from 10 series and 2560 patients from the literature [19]. Several nomograms and web or mobile phone applications are available to enable rapid risk category calculations to be made, which may assist multidisciplinary planning of patient management.

**Table 2:** Modified NIH risk classification for primary GIST

Risk category	Tumour size (cm)	Mitotic index (per 50 HPFs)	Primary tumour site
Very low risk	<2.0	≤5	Any
Low risk	2.1–5.0	≤5	Any
Intermediate risk	2.1–5.0	>5	Gastric
	<5.0	6–10	Any
	5.1–10.0	≤5	Gastric
High risk	Any	Any	Tumour rupture
	>10	Any	Any
	>5.0	>5	Any
	2.1-5.0	>5	Non gastric
	5.1–10.0	≤5	Non gastric

After Joensuu [18]

## Staging procedures

Staging procedures take into account the fact that most relapses affect the peritoneum and the liver. Contrast-enhanced abdominal and pelvic CT scan is the investigation of choice for staging and follow-up. Magnetic resonance imaging (MRI) or contrast-enhanced ultrasound may be alternatives, especially in younger patients where exposure to radiation should be limited. MRI provides better preoperative staging information for rectal GISTs. Chest CT scan or X-ray and routine laboratory testing complement the staging work-up of the asymptomatic patient, but are not routinely required during follow-up. Evaluation of ^18^F-fluorodeoxyglucose (FDG) uptake using an FDG-positron emission tomography (PET) scan, or FDG-PET–CT/MRI, can sometimes be useful, mainly when early assessment of response to tyrosine kinase inhibitor therapy is of special interest, for example after initiation of neo-adjuvant imatinib therapy.

## Treatment

When small oesophago-gastric or duodenal nodules less than 2 cm in size are detected, endoscopic biopsy may be difficult and laparoscopic/laparotomic excision may be the only way to make a histological diagnosis. Many of these small nodules, if diagnosed as GIST, will be low risk, or entities whose clinical significance remains unclear. Therefore, the standard approach to these patients is endoscopic ultrasound assessment, usually with fine needle aspiration or core needle biopsy, then annual follow-up, reserving excision for patients whose tumour increases in size or becomes symptomatic. Alternatively, the decision about a treatment plan can be discussed with the patient. This may depend on age, life expectancy and comorbidities. If follow-up is the choice, definitive evidence regarding an optimal surveillance policy is lacking, but annual follow-up is reasonable. For a small histologically proven GIST of 2 cm or greater, the standard treatment is excision, unless major morbidity is expected. Alternatively, in the case of a low-risk GIST, the possibility of surveillance could be discussed with the patient. Rectal (or recto-vaginal space) nodules should be biopsied and preferably excised after ultrasound assessment, regardless of tumour size. This is because GISTs at these sites have a higher risk of local recurrence after surgery, and the local implications for surgery in relation to morbidity are more critical. In specific clinical contexts, if the tumour is small, a follow-up policy without surgery could be adopted, but such an approach should be discussed in detail with the patient.

Multidisciplinary treatment planning is needed. This should involve histopathologists, radiologists, surgeons, and oncologists, as well as gastroenterologists, nuclear medicine specialists and others, as applicable. Such teams are available in reference centres for sarcomas and GISTs, which treat a large number of GIST patients annually. Support staff, such as clinical nurse specialists, play a vital role and are not likely to be available, or have the appropriate expertise, outside specialist centres.

### Localised disease—surgery

Standard treatment of localized GISTs is complete surgical excision of the lesion, with no dissection of clinically negative lymph nodes [III, A]. Surgery should be performed by a sub speciality surgeon who is fully trained and experienced in radical anatomic site specific cancer surgery. When adjacent organs are involved, *en bloc* resection is recommended wherever possible. If laparoscopic excision is planned, the technique needs to follow the principles of oncological surgery [20] [III, A]. A laparoscopic approach is clearly discouraged in patients with large tumours, because of the risk of tumour rupture, which is associated with a very high risk of relapse. R0 excision is the goal (i.e., an excision whose margins are clear of tumour cells). When R0 surgery is likely to result in major functional sequelae, e.g. total gastrectomy or abdomino-perineal resection of rectum, neo-adjuvant imatinib should be regarded as standard therapy [21–24] [IV, A]. Treatment is given to reduce the size of the tumour and to limit subsequent surgical morbidity. This may also be the case if surgery will be safer following cyto-reduction (e.g. the risk of bleeding and tumour rupture is likely to be decreased). After maximal tumour response, generally after 6–12 months, surgery is performed. Prior mutational analysis is crucial to prevent patients with less sensitive or resistant tumours (e.g. *PDGFRA* D842 V mutations) from receiving therapy with imatinib, and to allow appropriate dosing for patients with *KIT* exon 9 mutated tumours. Early tumour response assessment is mandatory, so that surgery is not delayed in the case of non-responding disease. Functional imaging, such as PET-CT, makes it possible to assess the tumour response very rapidly, within a few weeks. There are limited data to guide the physician on when to stop imatinib before surgery, but it can safely be stopped a few days, or even 1 day, before surgery and it can be resumed promptly when the patient has recovered from the acute effects of surgery.

If preoperative medical treatment has not helped or cannot be used, there should be a discussion with the patient about accepting a possible R1 resection with microscopically positive margins (i.e. excision margins containing tumour cells) [IV, B]. This may be more acceptable for low-risk lesions, with the lack of any formal demonstration that R1 surgery is associated with worse overall survival [25]. If an unplanned R1 excision has already been carried out, re-excision may be an option, provided the original site of lesion can be found, and major functional sequelae are not foreseen.

### Localised disease—adjuvant therapy

The risk of relapse following surgery can be substantial, as defined by available risk classifications. Adjuvant treatment with imatinib for 3 years was associated with improved relapse-free and overall survival compared with 1 year of therapy in a randomized trial in high-risk patients [26]. Previously, a placebo-controlled trial demonstrated that imatinib given for 1 year prolongs relapse-free survival in localized GISTs larger than 3 cm with a macroscopically complete resection [27]. Therefore, adjuvant therapy with imatinib for 3 years is standard treatment for patients with a significant risk of relapse [I, A] and was approved by the National Institute for Care and Health Excellence (NICE) in their recent re-appraisal (https://www.nice.org.uk/guidance/ta326). Mutational analysis is critical to making a clinical decision regarding adjuvant therapy. In fact, there is a consensus that *PDGFRA* D842 V-mutated GISTs should not be treated with any adjuvant therapy, given the lack of sensitivity of this genotype to imatinib both in vitro and in vivo [IV, A]. Given the data supporting the use of a higher dose of imatinib (800 mg daily) in the presence of an exon 9 *KIT* mutation in advanced GIST, clinicians might consider using this dose in the adjuvant setting for this genotype [28–31]. However, this is not supported by any controlled trial data in the adjuvant setting and use of the higher dose is not approved by NICE in the UK. There is consensus on avoiding adjuvant treatment in NF-1 related GISTs, which are insensitive to imatinib in the advanced setting. On the other hand, a consensus is lacking among experts about whether *KIT/PDGFRA* wild-type SDH-negative GIST should be treated with adjuvant therapy. This reflects their lower sensitivity to imatinib, as well as their peculiar natural history, which is often more indolent; subgroup analyses of available randomized trials are too limited to provide sufficient evidence.

If there has been tumour rupture before or during surgery, there will have been spillage of tumour cells into the peritoneal cavity, and therefore occult peritoneal disease can be assumed to exist. This puts the patient at a very high risk of peritoneal relapse. Therefore, these patients should be considered for adjuvant imatinib therapy. The optimal duration of treatment in these cases is unknown, given the uncertainty as to whether they should be viewed as essentially having metastatic disease, but should be at least 3 years, as for high risk resected GIST.

#### Key recommendations

1. GIST should be managed by an experienced multidisciplinary team.
2. Pre-operative imatinib should be considered for those large gastric or rectal primaries where immediate resection is likely to be morbid, e.g. total gastrectomy or abdomino-perineal resection. In this situation mutational analysis is mandatory prior to the initiation of imatinib therapy.
3. Patients at high risk of recurrence or distant relapse should receive 3 years of adjuvant imatinib, provided their tumour is not likely to be resistant to therapy (*PDGFRA* exon 18 mutation D842V).

### Metastatic disease—systemic treatment

In patients with inoperable and metastatic disease, imatinib is the standard treatment [32, 33] [III, A], including patients who had previously received the drug as adjuvant therapy without relapse during this treatment. This also applies to metastatic patients whose disease has been completely removed surgically, although surgery as a primary approach to metastatic GIST is not recommended. The standard dose of imatinib is 400 mg daily [I, A]. However, data have shown that patients with *KIT* exon 9 mutations fare better in terms of progression-free survival (PFS) on a higher dose of 800 mg daily, which is therefore the standard treatment in this subgroup [31] [III, A], albeit not recommended by a NICE appraisal which only assessed dose escalation in the context of disease progression. A report on the long term follow-up of the European/Australasian clinical trial which compared 400 mg with 800 mg imatinib in patients with advanced GIST has shown a survival advantage for the initial use of the 800 mg dose in those with exon 9 mutations in *KIT* (Casali P et al., in press) indicating a need to review this issue in the UK. Treatment should be continued indefinitely, since treatment interruption is generally followed by relatively rapid tumour progression, even when lesions have previously been surgically excised [34] [II, B]. At the start of treatment the patient should be alerted to the importance of adherence to therapy, and of possible interactions with concomitant medications and foods. They should also be given guidance about the best ways to handle any possible side effects. Dose intensity should be maintained by effective management of side effects, and a rational policy of dose reductions and interruptions should be applied if there is excessive, persistent toxicity. Retrospective data suggest that suboptimal plasma levels of imatinib are associated with a worse outcome, though a correlation with outcome has not been established prospectively [35]. A recent report confirmed that patients with imatinib trough levels of less than 760 ng/ml, taken after a minimum of 3 months’ treatment, which equates to steady state [36], had a worse outlook in terms of progression-free survival, which applied in the case of both gastric and small bowel GIST [37]. Aside from its potential use to tailor the imatinib dose, plasma level assessment may be useful in the case of: (i) patients receiving concomitant medications that put them at a risk of major interactions; (ii) unexpected observed toxicities; (iii) progression on 400 mg. Dose adaptation according to inadequate imatinib trough level is being studied in the Netherlands, and is a standard approach in a number of institutions. However, the use of a higher dose of imatinib in patients with progressive disease is not approved by NICE.

Close monitoring of the tumour response should be carried out in the early phases of treatment. Follow-up should be continued throughout the treatment, since the risk of secondary progression persists over time. Complete excision of residual metastatic disease has been shown to be related to a good prognosis, provided the patient is responding to imatinib, but whether this is due to surgery or to patient selection [38–40] has never been demonstrated prospectively. Conducting a randomised trial did not prove feasible; thus, at the present time surgery can be discussed with the patient but not recommended on the basis of a definitive proof of benefit [III, C]. Surgical excision of progressive disease is not recommended, given the poor results in published series, but surgery of limited progression, such as the ‘nodule within a mass’, has been associated with a progression-free interval in the same range as for second-line treatment with sunitinib. So, this may be a palliative option in the individual patient with limited progression, while continuing imatinib [V, C]. Non-surgical procedures, such as radiofrequency ablation of liver metastases may also be used. Prior to performing such interventions PET-CT can be useful to confirm the location of imatinib-resistant disease.

Dose escalation of imatinib to 800 mg in the case of a GIST with a *KIT* exon 9 mutation showing disease progression could be considered if the higher dose was not used initially, since the higher dose is significantly more effective in this setting [31]. Higher doses, though not necessarily 800 mg, could be useful if satisfactory plasma levels of imatinib are not being achieved, but the use of higher doses is not approved by NICE. The potential misinterpretation of the images produced by the complex tumour response patterns to TKIs can lead to a false diagnosis of progression, which must be considered. Patient non-compliance and drug interactions with concomitant medications must also be ruled out as the possible cause of progression.

If there is confirmed progression, or rare intolerance to imatinib after all attempts to manage side effects have failed, the standard second-line treatment is the tyrosine kinase inhibitor (TKI) sunitinib [41] [I, B]. This drug was proven to be effective in terms of PFS using a regimen of 50 mg daily 4 weeks on/2 weeks off. Data have been published showing that continuous treatment with a lower daily dose of 37.5 mg is also effective and well tolerated, although no formal comparison has been performed within a randomized clinical trial. This schedule can therefore be considered an alternative on an individualized basis [42] [III, B]. However, not all patients resistant to imatinib respond to sunitinib particularly those with secondary mutations affecting the activation loop domain of KIT and the *PDGFRA* exon 18 D842V mutation, which is always resistant.

A prospective placebo-controlled randomized trial demonstrated that regorafenib, at a dose of 160 mg daily on a 3 weeks on/1 week off schedule, significantly prolonged PFS in patients progressing after both imatinib and sunitinib [43]. Regorafenib is regarded as standard therapy for the third-line treatment of patients progressing on or failing to respond to imatinib and sunitinib [I, B]. It is currently available in England via the National Cancer Drugs Fund and is also available in Scotland, Wales and Northern Ireland as standard 3rd line therapy. The key distinction between sunitinib and regorafenib, as also previously shown with the analogue sorafenib, is its ability to inhibit tumours with secondary mutations in the activation loop of KIT, especially in exon 17 [44, 45]. These mutations are known to confer resistance both to imatinib and sunitinib, hence the value of regorafenib in this setting.

Patients with metastatic GIST failing all three standard agents should be considered for participation in clinical trials of new agents. These studies are only likely to be available in major centres treating GIST. There is limited evidence that patients who have already progressed on imatinib may benefit for a limited period when re-challenged with the drug [46]. Likewise, there is anecdotal evidence that maintaining treatment with a TKI even in the case of progressive disease, as opposed to stopping it, may slow down progression if no other option is available at the time. Therefore, re-challenging or continuing treatment with a TKI, to which the patient has already been exposed, is an option which may be considered for symptom control in patients with progression [V, B].

### Metastatic disease—local therapy

Selected patients with limited liver metastatic disease may be amenable to surgery or radiofrequency ablation (RFA) after maximum response to imatinib, or if there is evidence of localised disease progression. The use of RFA is restricted to tumours in the region of 3 cm in maximum diameter and is less likely to be a suitable approach for lesions adjacent to large vessels or superficial lesions, especially if displacing the liver capsule. However, larger isolated lesions and superficial lesions may still be suitable for surgical resection, either by partial hepatectomy or wedge resection. Dedicated liver MRI scans and when appropriate PET-CT scans may be required to determine whether this is a legitimate approach by excluding other occult active disease.

Radiotherapy can be a useful local therapy in GIST under certain circumstances in the advanced disease setting. If there is a single site of disease that is progressing on a TKI and can be encompassed within a radiotherapy treatment field, then radiotherapy delivered to a moderate or high dose can offer local tumour control, and possibly prolong the use of the TKI [47]. Radiotherapy can also be used at lower doses to palliate symptomatic disease, for example to relieve pain or bleeding.

### Response assessment

Response assessment is complex and early progression in particular should be confirmed by a team experienced in treating GIST. Anti-tumour activity translates into tumour shrinkage in most patients, but some patients may show only changes in tumour “density” on imaging, these changes sometimes precede a reduction in tumour volume. Such changes in tumour radiological appearance should be considered as indicative of tumour response. Tumour size may even increase in the short term but if tumour density on CT scan is decreased this may still indicate tumour response [48, 49]. Even the apparent ‘appearance’ of new lesions may be due to them becoming less dense, or cystic, especially in the liver. Therefore, both tumour size and tumour density on CT scan, or consistent changes on MRI or contrast-enhanced ultrasound, should be considered when determining tumour response. ^18^F-FDG-PET has proved useful in the early assessment of tumour response, for example when prediction of the response is valuable, for example in the case of preoperative therapy, or when response is in doubt. However, a small proportion of GISTs have no FDG uptake. The absence of tumour progression at 6 months [50] is also equivalent to a tumour response. Conversely, tumour progression may not always be accompanied by changes in tumour size. For example, an increase in the tumour density shown by contrast enhancement within a previously responding low density tumour lesion, may be indicative of tumour progression. A typical progression pattern is the ‘nodule within the mass’, in which a portion of a responding lesion becomes hyper-dense [51].

#### Key recommendations

1. Imatinib is the treatment of choice for patients with unresectable or metastatic disease and is given until progression at the standard dose of 400 mg daily. Data suggest that patients whose tumours have an exon 9 mutation in *KIT* benefit from a larger dose, though this is not currently recommended by NICE.
2. Isolated progression may be amenable to surgery or other local measures, such as radiofrequency ablation.
3. Standard second line treatment is sunitinib, which may be given at the recommended dose of 50 mg daily for 4 weeks every 6 weeks, or 37.5 mg daily continuously.
4. Standard 3rd line treatment is regorafenib.

## Follow-up

The optimal follow-up policy for surgically treated patients with localized disease is unclear. Relapses occur most often in the liver and/or the peritoneal cavity. Other sites of metastases, including bone and brain are uncommon, but may be less unusual following prolonged treatment with several lines of therapy. The mitotic rate most likely affects the frequency with which relapses occur. Risk assessment based on the mitotic count, tumour size and tumour site may be useful in choosing the routine follow-up policy. High-risk patients generally relapse within 1–3 years from the end of adjuvant therapy. Low-risk patients may relapse later, given that the disease is likely to be slower growing.

The issue of follow-up has been addressed by Joensuu and colleagues based on the currently available evidence [52]. High-risk patients who have undergone resection of their primary generally undergo a routine follow-up with abdominal CT or MRI scan every 3–6 months during adjuvant therapy, for 3 years. This frequency of follow up is because of the need to manage the side effects of the therapy. On cessation of adjuvant therapy follow up is every 3 months for 2 years, then every 6 months for another 3 years, after which follow up is annually for another 5 years. Patients with high-risk tumours not given adjuvant therapy, for whatever reason, should be followed up 3 monthly for 2 years, 6 monthly for 3 years and then annually for a further 5 years.

For low to intermediate risk tumours, the optimal frequency of follow-up is less clear. If follow-up is performed, it will usually be an abdominal CT or MRI scan, or ultrasound, every 6–12 months for 5 years.

Very low-risk GISTs do not require routine follow-up, provided excision was complete, although one must be aware that the risk of progression is not zero.

Radiation exposure is a factor to consider when selecting the imaging modality for long term follow-up. Abdominal MRI is an acceptable alternative to CT which could be used at selected intervals.

### Key recommendations

1. Patients with high risk disease on adjuvant therapy should be followed up by cross-sectional imaging every 3–6 months during their 3 years of treatment, 3 monthly for 2 years following cessation of treatment and thereafter every 6 months for 3 years and annually for 5 years.
2. Patients with high risk disease not receiving adjuvant treatment should follow the post-adjuvant treatment scheme.
3. For patients with lesser levels of risk less frequent follow-up is generally recommended, although the clinical benefits are unclear. For intermediate risk patients 6 monthly scans for 5 years followed by annual scans and for low risk patients shorter duration follow-up would be considered reasonable.
